# Evidence for Patterns of Selective Urban Migration in the Greater Indus Valley (2600-1900 BC): A Lead and Strontium Isotope Mortuary Analysis

**DOI:** 10.1371/journal.pone.0123103

**Published:** 2015-04-29

**Authors:** Benjamin Valentine, George D. Kamenov, Jonathan Mark Kenoyer, Vasant Shinde, Veena Mushrif-Tripathy, Erik Otarola-Castillo, John Krigbaum

**Affiliations:** 1 Department of Anthropology, Dartmouth College, Hanover, NH, United States of America; 2 Department of Geological Sciences, University of Florida, Gainesville, FL, United States of America; 3 Department of Anthropology, University of Wisconsin—Madison, Madison, WI, United States of America; 4 Department of Archaeology, Deccan College Post-Graduate and Research Institute, Pune, Maharashtra, India; 5 Department of Human Evolutionary Biology, Harvard University, Cambridge, MA, United States of America; 6 Department of Anthropology, University of Florida, Gainesville, FL, United States of America; New York State Museum, UNITED STATES

## Abstract

Just as modern nation-states struggle to manage the cultural and economic impacts of migration, ancient civilizations dealt with similar external pressures and set policies to regulate people’s movements. In one of the earliest urban societies, the Indus Civilization, mechanisms linking city populations to hinterland groups remain enigmatic in the absence of written documents. However, isotopic data from human tooth enamel associated with Harappa Phase (2600-1900 BC) cemetery burials at Harappa (Pakistan) and Farmana (India) provide individual biogeochemical life histories of migration. Strontium and lead isotope ratios allow us to reinterpret the Indus tradition of cemetery inhumation as part of a specific and highly regulated institution of migration. Intra-individual isotopic shifts are consistent with immigration from resource-rich hinterlands during childhood. Furthermore, mortuary populations formed over hundreds of years and composed almost entirely of first-generation immigrants suggest that inhumation was the final step in a process linking certain urban Indus communities to diverse hinterland groups. Additional multi disciplinary analyses are warranted to confirm inferred patterns of Indus mobility, but the available isotopic data suggest that efforts to classify and regulate human movement in the ancient Indus region likely helped structure socioeconomic integration across an ethnically diverse landscape.

## Introduction

Protohistoric South Asia holds unique insights into the evolution and maintenance of early urbanism, as the relatively decentralized Indus Civilization suggests an alternative to the strongly centralized states of contemporaneous Egypt and Mesopotamia [1]. Yet the institutional mechanisms that structured Indus expansion in the late 3^rd^ millennium BC remain unclear, in part because the Indus script is undeciphered. Fortunately, human tooth enamel provides a biogeochemical archive of past behavior and residence change that offers an alternative means of reconstructing ancient institutions [2]. The nature of the Indus skeletal record suggests that Indus cemetery inhumations are closely associated with the socio-political structures of the Harappa Phase (2600–1900 BC) and therefore provide a key source of isotopic data for understanding early urban mechanisms of interaction. Like standardized weights, measures, script, and stamp seals, a relatively homogenous tradition of cemetery inhumation endured for centuries, contemporaneous with the Indus urban florescence and cultural integration spanning ~680,000 km^2^ of northwest South Asia [3]. Though burials are geographically widespread, more than a century of excavation has yielded skeletal remains for only ca. 600 individuals [4]. Inhumations in formalized cemetery contexts are very rare, suggesting that cemetery populations represent a specific group distinct from the population-at-large. In this work, we conducted lead (Pb) and strontium (Sr) isotopic analysis of human tooth enamel recovered from Harappa Phase cemeteries at Harappa, Pakistan [5] and Farmana, India [6] in order to assess the dynamics of migration and social identity for the buried individuals. We demonstrate how the isotopic data can be used to elucidate their distinctive social identity and propose that a specific, culturally regulated institution of migration helped to shape ancient urban-hinterland relationships.

### Indus Mortuary Practice in Context

**Fig 1:**
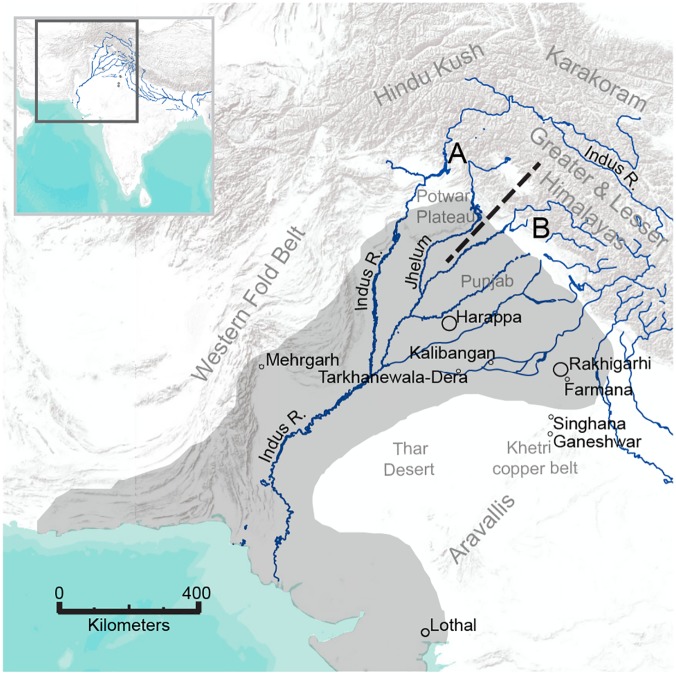
Map of the Indus Civilization culture area with locations mentioned in the text. Dashed line indicates approximate boundary between geochemical catchments. Catchment A, including the Potwar Plateau and adjacent drainages of the Hindu Kush and Karakoram, is relatively less radiogenic than Catchment B, including the Punjab tributaries that drain the Greater and Lesser Himalayas.

Though peoples of the Indus Tradition were connected by trade routes since at least 5500 BC, it was not until ca. 2600 BC that diverse regional cultures developed a common repertoire of ceramic forms, settlement organization, urban infrastructure, and administrative practices [1, 7]. Emerging elites sought to consolidate their advantages through the control of exotic resources and sophisticated craft technologies [8]. At the major urban center of Harappa, for example, mercantile groups acquired a wide range of resources from adjacent mineral-rich regions including the Potwar Plateau to the northwest [9] (Fig 1). Farmana was a much smaller settlement near the eastern limit of the Indus culture area and relatively close to the Khetri copper belt of the northern Aravalli mountain range, a region known for ancient copper production [10]. Indus peoples likely acquired raw materials from these and other regions through trade, as there is little archaeological evidence for large-scale militarization [9]. Furthermore, Indus artifacts and stylistic influences at hinterland sites suggest relationships of reciprocity [9, 11].

Mortuary analysis of Harappa Phase cemetery populations complements approaches to understanding interregional interaction that focus primarily on the movement of artifacts. As burials are rare, the vast majority of Indus deceased were presumably disposed of in ways that are not apparent in the archaeological record. However, important insights have been gleaned from available mortuary populations. Previous morphological and strontium isotope studies of skeletal material at the sites of Harappa [12, 13] and Lothal [14] suggest residence change may have been common for certain individuals and that increased mobility facilitated gene flow with hinterland groups. At Harappa, males show greater isotopic and morphological variation—a pattern previously interpreted as evidence for matrilocality [13]. Likewise, morphological similarity between individuals from Lothal and nearby hunter-gatherers suggests some degree of urban-hinterland mobility [14].

Despite the potential influx of immigrants, Harappa Phase burials are relatively homogenous in terms of material culture and contain artifacts known from domestic Indus contexts [7]. Most Indus cemetery burials consist of a rectangular or oval pit aligned north to south with modest numbers of associated ceramics at the head of the grave. Skeletal remains typically lie supine and extended and are sometimes adorned with small quantities of jewelry or other personal effects. Skeletons may be incomplete or absent with grave goods present in various quantities, but nearly all cemetery inhumations conform to a similar layout that is not readily differentiated into distinct social classes.

The skeletal series from Harappa (Cemetery R-37) and Farmana are consistent with the above trends, and we suggest the inhumed participated in a shared set of mortuary practices. These practices remained relatively stable over a period of several centuries as indicated by radiocarbon dates from charcoal associated with early and late burials at Harappa (2550–2030 cal BC) (J. M. Kenoyer, personal communication). Relative dating from ceramics associated with the Farmana burials indicates a similar period of use (ca. 2600–2000 BC) [6, 15]. Few clues exist as to the social identity of the deceased, but the low incidence of skeletal pathologies and non-specific stress indicators at Cemetery R-37 suggests improved access to resources [13]. Likewise, personal ornaments are few in number but many are of high quality [5]. Although Harappa Phase burials lack the kinds of ostentatious display associated with royal Mesopotamian and Egyptian tombs, their relatively privileged disposition in life and death could indicate ties with elite Indus groups. Lastly, demographic profiles of the Harappa Phase mortuary populations at Harappa [5] and Farmana [6] further emphasize the selective nature of the mortuary program. A near total absence of infants implies certain social strictures on burial. Given the frequency with which children receive alternative mortuary treatments cross-culturally [16, 17], the Indus demographic profiles cannot be interpreted as direct evidence for a distinct social class associated only with older individuals. Nevertheless, they are consistent with the notion that Harappa Phase mortuary populations are not wholly representative cross-sections of particular breeding populations or ethnic groups.

The question of who was incorporated into cemeteries has major implications for any mortuary analysis. For decades, archaeologists have looked to the ordering principles of mortuary populations for insights into broader social systems. In certain instances, patterns of intra-cemetery variability may provide relatively direct reflections of social hierarchies [18, 19, 20, 21]. However, this view has been critiqued by scholars emphasizing the ways in which mortuary programs are the subject of active manipulation and capable of influencing societies in their own right [22, 23, 24, 25]. Conceived in this way, archaeological cemeteries must be interpreted in terms of dynamic social processes embedded within particular historical contexts. A similar conceptual approach has guided the study of frontiers or borderlands [26, 27, 28], and indeed, it is not uncommon for novel mortuary programs to emerge in situations of culture contact [29, 30, 31, 32]. Therefore, isotopic methods for assessing the origins of individuals provide invaluable tools for inferring the presence and character of cultural interaction.

Isotopic approaches coupled with bioarchaeological methods permit a more robust mortuary analysis because they yield data at the level of the individual. This analytical resolution can be crucial for determining the structure of intra-population variation. However, the individual may not always be the most relevant unit of analysis when modeling social systems. In the context of a mortuary program, individual bodies should not be conflated with individual social identities because burial assemblages are not autobiographical. They are produced by the living, and each burial gives the living an opportunity to assert their own perceptions [23]. Burials offer a medium in which the living can emphasize diverse relationships with the deceased [33], or in some cases, with the deceased’s corporate group [34]. Therefore, models of social systems derived from mortuary data must consider the role of multi-bodied, multi-generational social identities in structuring mortuary practices. Given the relative homogeneity and extreme scarcity of Harappan Phase cemetery burials, it is possible that a common group identity was prioritized over various individual identities. Thus, the traits that burials share may yield more insights into the mortuary program than traits that differentiate one burial from another.

### Principles of Analysis

Combined analyses of radiogenic Sr and Pb isotope ratios (i.e., ^87^Sr/^86^Sr, ^208^Pb/^204^Pb, ^207^Pb/^204^Pb, ^206^Pb/^204^Pb) and Sr abundances in dental enamel samples enable more detailed inferences about Indus mobility and cultural integration. Each bulk enamel sample yields weighted average isotopic ratios of the bioavailable or soluble fraction of Sr and Pb ingested in food, water, and dust during the period of enamel mineralization [35, 36, 37]. Because ratios of these heavy isotopes remain unchanged as they pass from bedrock into the biosphere, isotopic differences between tooth enamel samples can be attributed to different environmental sources of Sr and Pb originating from distinct geographic regions. In other words, heavy isotope ratios in tooth enamel can be matched to those of the place (or places) where an individual was living. The same is potentially true for other mineralized tissues like bone and dentine, but the crystalline structure of enamel apatite is much more resistant to diagenetic alteration in the burial environment and provides a high-fidelity record of *in viv*o isotopic ratios [35, 36, 37].

Once fully mineralized, enamel undergoes no additional remodeling [38] and therefore archives the isotopic input acquired during a specific developmental period in an individual’s life history. Variations in the timing of enamel mineralization mean that early-life residential history can be inferred through analyses of multiple tooth types—grouped here into three cohorts. The first molar cohort consists of teeth that mineralized within the first three years of life, whereas second and third molar cohorts represent ~3–7 years of age and ~8–16 years of age, respectively [39, 40]. Though we present maximum age ranges for completeness, the mineralization of premolars and molars has been noted to take between 3.0 and 3.4 years [39]. Comparison of enamel isotope ratios from different molar cohorts with estimates of interregional isotopic variation permits inferences about the location and timing of individual residence changes [35, 36, 37]. Although variability in diet and residence over a multi-year period may prevent precise correlations between a given enamel sample and estimated isotopic catchments, patterning within an isotopic dataset relative to geologic end-members can support more detailed interpretations. In addition, mixing lines between distinct geological sources may be inferred using elemental concentration data, making it possible to distinguish between groups of individuals that have similar isotopic ratios but different elemental concentrations (i.e., Sr ppm) [41].

Individuals are identified as non-local when their isotope ratios fall outside the estimated range of isotopic variation for the local dietary catchment. Local water, sediment leachates, plants, and faunal remains can all be used as proxies for the bioavailable isotopic input in human dietary catchments, but none are likely to give a precise weighted average of the most frequently ingested sources of food, water, and dust in the human diet [42]. Tooth enamel from archaeological fauna for species with small home ranges provides the best estimate of local isotopic variation because foraging behavior averages out small-scale geochemical variations within a relatively constrained area. Sampling archaeological material also minimizes the potential influence of modern environmental contamination. Nevertheless, factors including seasonal migration or the importation of faunal resources can introduce non-local ratios into datasets which can result in overestimates of local isotopic variability.

To deal with the challenges of defining local isotopic ranges, Burton and Price [43] suggested using faunal ^87^Sr/^86^Sr to confirm the local modal trends in human ^87^Sr/^86^Sr as identified through kernel density estimation. We follow a similar logic in emphasizing the internal structure of the data sets, but apply a different statistical method for this multi-isotopic study. Here we use cluster analysis algorithms DBSCAN [44] and K-means [45]. Given some knowledge of underlying isotopic variability in the geological setting, such methods can discriminate between archaeological individuals within well-represented clusters (presumably frequently accessed dietary catchments) and those that are peripheral to the identified clusters.

## Materials and Methods

Whenever possible, teeth from the first, second, and third molar cohorts were sampled to maximize life history information for each individual. First molars were preferred for assaying early life isotopic exposure, but a single first incisor from Harappa burial H87/ 72 49h was included in the first molar cohort to maximize the dataset. Based on similar logic, three premolars were included in the second molar cohort. Even though initial cusp formation begins at about 1 year of age for canines, a single canine was also grouped with the second molar cohort for two reasons. First, canine crowns have a long formation time, lasting until ~5–6 years of age. Second, the sampled canine was clearly associated with the first incisor from burial H87/ 72 49h, a tooth that mineralized earlier in development. Together with teeth from burials H87/ 71 49c and H94/ 253 18, these samples provide the only developmental sequences that could be confirmed from the Harappa analyses. Unfortunately, many Harappa burials were either poorly preserved or had been disturbed in antiquity by Harappa Phase residents re-using cemetery land for subsequent burials [5]. All available fully mineralized teeth were sampled from collections at the Harappa Museum in Harappa, Pakistan yielding 44 teeth from a minimum number of individuals (MNI) of 38. The Farmana sample, curated at Deccan College Post-Graduate and Research Institute in Pune, India, includes 33 teeth from 17 individuals.

Additional faunal and sedimentary samples were collected to establish a baseline for local isotopic variation. Sampled archaeological fauna include pigs (*Sus*) and dogs (*Canis*), all of which were collected from secure chronological contexts at Harappa (n = 13) and Rakhigarhi (n = 8). Farmana fauna were unavailable for sampling, but the major urban center of Rakhigarhi lies ~30 km to the northwest of Farmana and is assumed to be broadly representative of the bioavailable dietary catchment in the region. Sediment samples (~200 mg) collected at Farmana (n = 3) augment the Rakhigarhi faunal data. Harappa and Rakhigarhi fauna are curated at the Peabody Museum in Cambridge, USA and Deccan College respectively. Permission to export samples for isotopic analysis was granted by the Archaeological Survey of India. All necessary permits were obtained for the described study, which complied with all relevant regulations.

Tooth enamel samples (~50 mg) were cleaned of surficial deposits and dentine under 10x magnification using a Brassler dental drill with a diamond bit. Samples were powdered in agate mortars and pestles and pretreated according to light stable isotope protocols [46] using solutions of 50% reagent grade sodium hypochlorite and 0.2 N acetic acid. Approximately 10 mg of each pretreated enamel sample was set aside for light stable isotope analyses not reported here, and the remainder was reserved for heavy isotope analyses in the clean lab and mass spectrometry facilities of the Department of Geological Sciences, University of Florida. Sediments were leached for 2 hour periods using 0.1 N acetic acid and 2 N hydrochloric acid successively, with each acid leachate pipetted off to capture the bioavailable fraction of Sr and Pb. All enamels and sediment leachates were dissolved in pre-cleaned Teflon vials using 8 N nitric acid, after which Sr and Pb were separated from single aliquots using ion chromatography [47].

All samples were analyzed for ^87^Sr/^86^Sr using a “Micromass Sector 54” thermal ionization mass spectrometer (TIMS). After being loaded onto degassed tungsten filaments, the samples were run at 1.5V for 100 ratios whenever possible, and the resulting data normalized to ^86^Sr/^88^Sr = 0.1194. The long-term reproducible ^87^Sr/^86^Sr of NBS-987 is 0.710240 ± 0.000023 (2σ). Lead isotopic ratios were measured using a ‘‘Nu-Plasma” multiple-collector inductively-coupled-plasma mass spectrometer (MC-ICP-MS) with Tl-normalization [48]. The data are reported relative to the following values of NBS 981: ^206^Pb/^204^Pb = 16.937 ± 0.004 (2σ), ^207^Pb/^204^Pb = 15.490 ± 0.003 (2σ), and ^208^Pb/^204^Pb = 36.695 ± 0.009 (2σ). Additionally, sample preparation, Sr and Pb separation, and TIMS and MC-ICP-MS analyses were conducted in multiple sessions over a period of several months with each session incorporating samples from multiple tooth types. As a consequence, systematic patterns within the dataset are not attributable to errors in the analytical process.

Statistical analyses were used to identify optimal clusters in the faunal isotopic distributions (^206^Pb/^204^Pb vs. ^87^Sr/^86^Sr) from Harappa and Rakhigarhi, following the assumption that the modality of the results (i.e., the most frequently returned number of clusters) reflects the underlying structure of the data set and the most plausible cluster size. We used the DBSCAN algorithm to assign data points to clusters according to density reachability. DBSCAN requires the operator-defined parameters *minpts* (minimum number of points needed to form a cluster) and *ε* (maximum radius to consider when determining whether two points are directly density-reachable) [44]. DBSCAN analysis was conducted on the normalized data from each site (*minpts* = 1) with *ε* defined at 1000 regular sequential intervals for the maximum range of values that returned non-trivial results. Upper and lower bounds were defined as the highest and lowest values of *ε* that neither assigned each data point to its own unique cluster nor grouped all data within a single cluster (Harappa: 0.214 ≤ *ε* ≤ 2.069, Farmana: 0.235 ≤ *ε* ≤ 1.706). We then identified the optimal clustering solution for each site using maximum-likelihood estimation under the Poisson model [49]. Clustering solutions with the highest likelihood were selected as representative of the local isotopic range. Local isotopic ranges were defined as the minimum and maximum ratios for all data points within the largest cluster of the most likely clustering solution.

DBSCAN analysis was compared against results from the K-means clustering algorithm [45]. K-means analysis was conducted for sequential values of *k* (the number of possible clusters) with the optimal solutions for *k* (those which explain most of the variance with the fewest clusters) identified at the “elbow” in scree plots of the sum square of errors (SSE). Replication of the dense central clusters identified in DBSCAN lends supports to the distinction between clustered “local” data points and peripheral clusters or data points. All analyses were conducted using R, version 3.1.1 [50] and the fpc package [51].

## Results

Results of the individual analyses are reported in S1 Table with means (M), standard deviations (SD) and ranges provided below. At Harappa, human ^87^Sr/^86^Sr (M = 0.71623, SD = 0.00378) has a wider range (0.71113–0.72802) than that of fauna (M = 0.71865, SD = 0.00135, range = 0.71569–0.72112). The same is true for Harappa human Pb isotope ratios [^208^Pb/^204^Pb (M = 38.814, SD = 0.257, range = 38.013–39.377), ^207^Pb/^204^Pb (M = 15.713, SD = 0.026, range = 15.650–15.770), ^206^Pb/^204^Pb (M = 18.677, SD = 0.171, range = 18.054–19.099)] relative to those of Harappa fauna [^208^Pb/^204^Pb (M = 38.970, SD = 0.159, range = 38.580–39.189), ^207^Pb/^204^Pb (M = 15.735, SD = 0.008, range = 15.720–15.749), ^206^Pb/^204^Pb (M = 18.744, SD = 0.106, range = 18.468–18.874)]. A similar relationship holds for Farmana humans [^87^Sr/^86^Sr (M = 0.71611, SD = 0.00111, range = 0.71529–0.72038), ^208^Pb/^204^Pb (M = 38.921, SD = 0.606, range = 36.279–39.413, ^207^Pb/^204^Pb (M = 15.755, SD = 0.050, range = 15.572–15.814), ^206^Pb/^204^Pb (M = 18.897, SD = 0.567, range = 16.506–19.896)] compared to Farmana sediment leachates [^87^Sr/^86^Sr (M = 0.71565, SD = 0.00015, range = 0.71553–0.71594), ^208^Pb/^204^Pb (M = 39.424, SD = 0.024, range = 39.393–39.452), ^207^Pb/^204^Pb (M = 15.819, SD = 0.004, range = 15.813–15.823), ^206^Pb/^204^Pb (M = 19.328, SD = 0.020, range = 19.297–19.343)] and Rakhigarhi fauna [^87^Sr/^86^Sr (M = 0.71618, SD = 0.00131, range = 0.71471–0.71903), ^208^Pb/^204^Pb (M = 38.949, SD = 0.256, range = 38.371–39.188), ^207^Pb/^204^Pb (M = 15.752, SD = 0.046, range = 15.655–15.799), ^206^Pb/^204^Pb (M = 18.882, SD = 0.169, range = 18.584–19.053)]. Furthermore, the Harappa human isotope data have broadly distinct distributions from the Farmana human data set. This observed difference is also apparent in measured Sr concentrations (Harappa Sr ppm—M = 332, SD = 88, range = 137–557; Farmana Sr ppm—M = 826, SD = 246, range = 246–1388).

**Fig 2:**
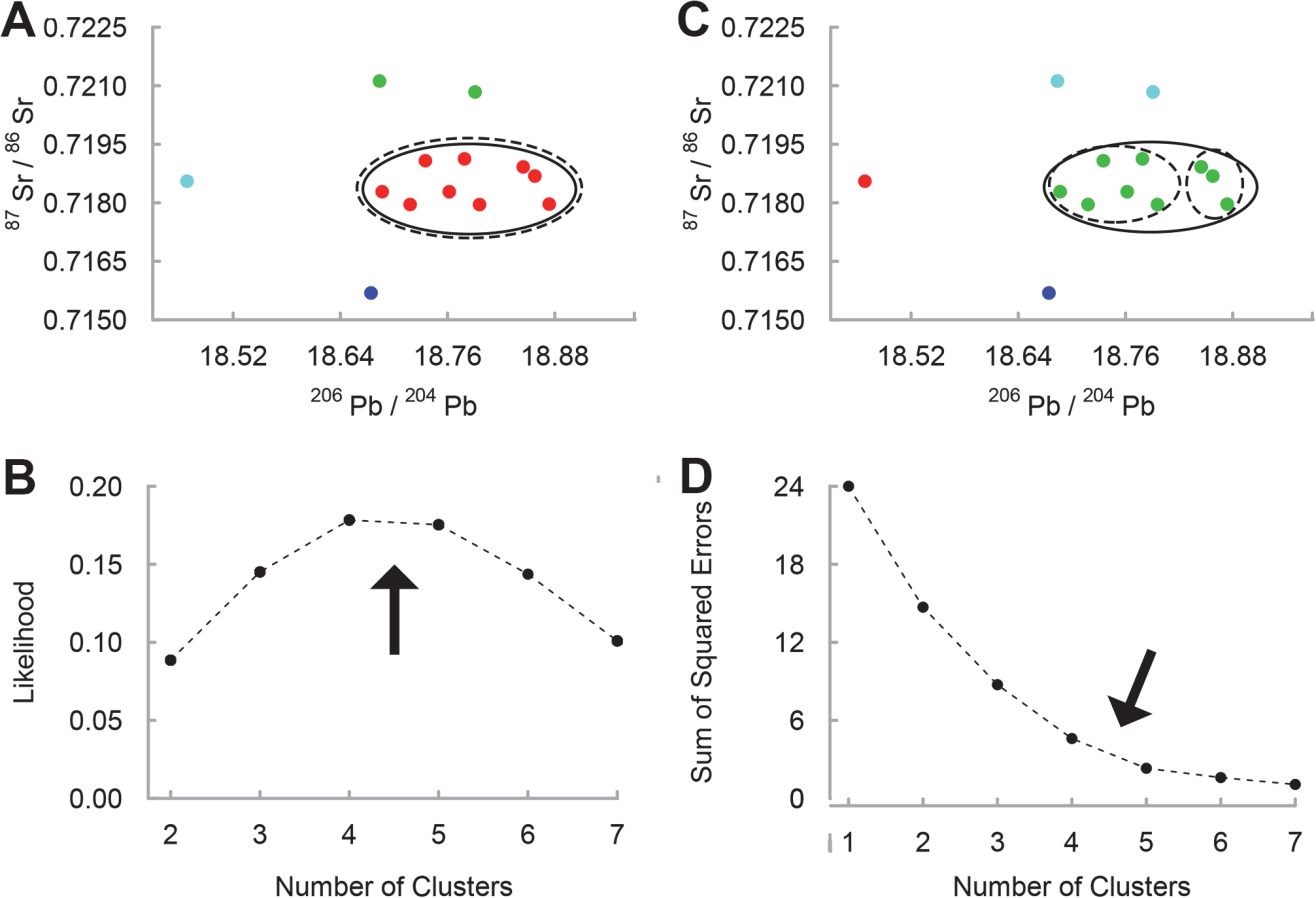
Local dietary catchment at Harappa inferred through cluster analysis of faunal isotope ratios. The structures of the optimal clustering solutions are depicted in Pb-Sr space (^206^Pb/^204^Pb vs. ^87^Sr/^86^Sr) with each cluster assigned a different color. Solid circles identify the local faunal cluster determined by the four-cluster solutions, dashed circles identity the local faunal cluster determined by the five-cluster solutions. The (A) optimal DBSCAN clustering solution in Pb-Sr space is inferred from (B) a likelihood estimation of all non-trivial DBSCAN clustering solutions. The (C) optimal K-means clustering solution is inferred from (D) the “elbow” in the K-means SSE plot.

**Fig 3:**
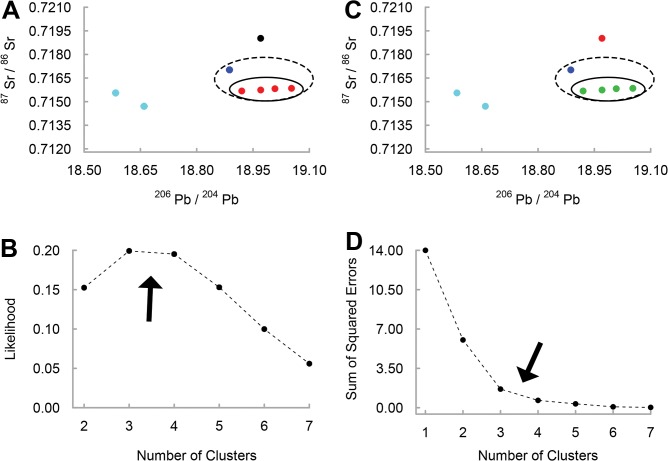
Local dietary catchment at Rakhigarhi inferred through cluster analysis of faunal isotope ratios. The structures of the optimal clustering solutions are depicted in Pb-Sr space (^206^Pb/^204^Pb vs. ^87^Sr/^86^Sr) with each cluster assigned a different color. Solid circles identify the local faunal cluster determined by the four-cluster solutions, dashed circles identity the local faunal cluster determined by the three-cluster solutions. Arrows indicate optimal clustering solutions. The (A) optimal DBSCAN clustering solution in Pb-Sr space is inferred from (B) a likelihood estimation of all non-trivial clustering solutions. The (C) optimal K-means clustering solution is inferred from (D) the “elbow” in the K-means SSE plot.

Cluster analysis of the faunal data reveals the structure of their distributions in Pb-Sr space and suggests plausible ranges for defining local isotopic variability (Figs 2 and 3). Site-specific analyses in DBSCAN yield clusters of varying size for different values of *ε*. For Harappa fauna, the clustering solutions with highest likelihood consist of four (0.178) and five (0.175) clusters (Fig 2B). In both solutions, the large central cluster is composed of the same nine data points (Fig 2A). The nine-point cluster is further validated by K-means analysis. Fig 2D shows that SSE is minimized as the number of clusters is increased, but the rate of minimization levels off rapidly (forming the “elbow”) beyond the four and five cluster solutions. The largest cluster in the four cluster solution mirrors that produced by DBSCAN (Fig 2C), whereas the five cluster solution subdivides the central cluster into two smaller subclusters based on variation in ^206^Pb/^204^Pb. Thus, both DBSCAN and K-means analyses support the partitioning of the data set into a central cluster and peripheral clusters or data points. Furthermore, the balance of evidence identifies a nine-point cluster having a ^87^Sr/^86^Sr SD of 0.0005. This value is broadly consistent with the typical SD of 0.0003 proposed by Burton and Price [43], and the relatively larger SD inferred here for the major urban center of Harappa might be attributable to the proportionately larger provisioning area exploited by Harappan residents. We therefore define the local range at Harappa as the minimum and maximum of ^87^Sr/^86^Sr (0.71795–0.71913) and ^206^Pb/^204^Pb (18.687–18.874) for the nine-point faunal cluster (S2 Table).

Similar partitioning is apparent within the Rakhigarhi fauna. Using DBSCAN, the most likely clustering solutions consist of three (0.199) and four (0.195) clusters (Fig 3B) resulting in primary clusters of five and four points respectively (Fig 3A). K-means analysis identifies the same clusters (Fig 3C) as outcomes of the three and four cluster solutions inferred from the elbow of the scree plot (Fig 3D). The four-point cluster has a slightly smaller ^206^Pb/^204^Pb SD (0.056) compared to that of the five-point cluster (0.066), but a striking difference is apparent in ^87^Sr/^86^Sr. In the four-point cluster, all the data points fall between 0.7157 and 0.7158 (SD = 0.0001), whereas the five-point cluster (SD = 0.0006) includes a far more radiogenic data point (0.7170). Although ^87^Sr/^86^Sr variability for the five-point cluster is more comparable with that of Harappa, it is not consistent with either the known ^87^Sr/^86^Sr homogeneity in the region [52] or the distribution of the Farmana data set. All but four of the Farmana human samples have ^87^Sr/^86^Sr at or below 0.7160. These values contrast markedly with the relatively radiogenic data point from the five-point faunal cluster. Likewise, the sediment leachates from Farmana have ^87^Sr/^86^Sr more similar to that of the four-point faunal cluster (0.7155–0.7159). Lastly, radiogenic isotope data should be distributed normally for a given population exploiting a single dietary catchment [43]. A Shapiro-Wilk normality test was significant (p = 0.004) only for the five-point cluster, supporting an inference of non-normality, which is inconsistent with a single dietary catchment. Therefore, we suggest that the four-point cluster best represents the local isotopic range at Rakhigarhi. For purposes of inferring migration in the Farmana mortuary population, we define the local range at Farmana as the minimum and maximum of ^87^Sr/^86^Sr (0.71553–0.71594) and ^206^Pb/^204^Pb (18.920–19.343) for the pooled sample of Farmana sediment leachates and the four-point Rakhigarhi faunal cluster (S2 Table).

## Discussion

### Defining Local

Isotopic analyses of humans and fauna show considerable variability consistent with changes in residence. Although human isotopic ranges exceed those of the fauna, wide variability in the faunal isotopic ratios nevertheless suggests that they overestimate local isotopic catchments. For example, the standard deviation of ^87^Sr/^86^Sr for all fauna at Harappa (0.0014) far exceeds the “typical” SD of approximately 0.0003 for archaeological sites [43]. Likewise, the relatively large SD at Rakhigarhi (0.0013) is inconsistent with the ^87^Sr/^86^Sr homogeneity known for regional sediments in general [52] and Farmana sediments (SD = 0.0002) in particular. Given that biological samples tend to show less variation than geological samples due to averaging effects [42], the relatively narrow isotopic distribution of Farmana sediment leachates suggests that Rakhigarhi faunal variation exceeds that of the local dietary catchment. The best explanation for faunal isotopic variability at Harappa and Rakhigarhi is that some of the sampled fauna had non-local origins. Although imported foodstuffs could theoretically produce non-local Sr isotope ratios in local populations, food is an unlikely source of Pb isotope variability given the importance of particulate in controlling biological Pb burdens [37]. Indeed the occasional long-distance transport of faunal resources such as pigs is hardly surprising given the extensive overland and riverine trade routes of the Indus Civilization [53].

**Fig 4:**
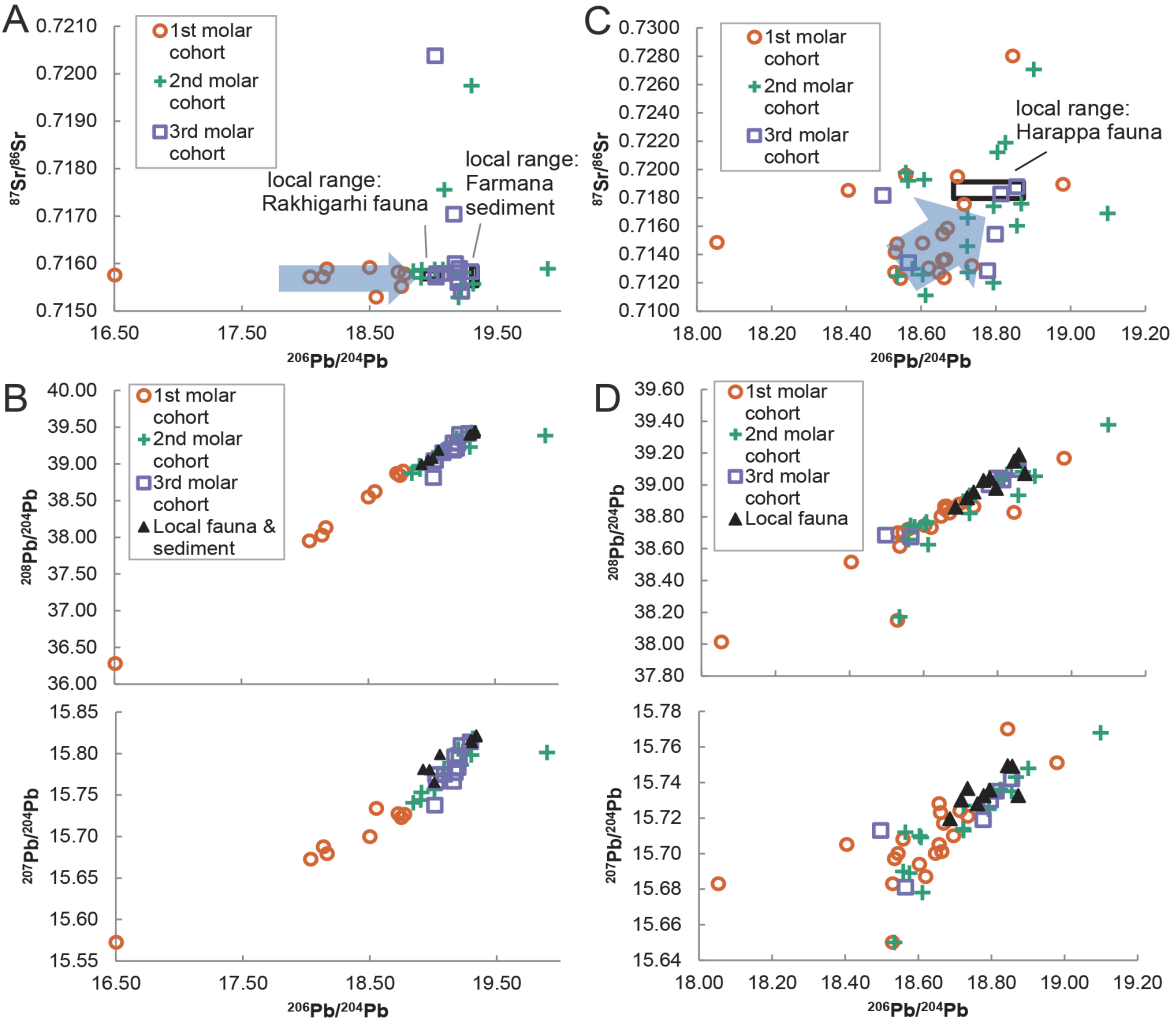
Heavy isotope ratio scatter plots of Indus Civilization human tooth enamel. Data are sorted by tooth type and plotted against inferred local ranges (indicated by black boxes). Arrows emphasize the broad progression from non-local first molars to local third molars. Relative to local sediment leachates and Rakhigarhi fauna, Farmana first molars have non-local distributions in (A) Pb-Sr space (^206^Pb/^204^Pb vs. ^87^Sr/^86^Sr) and (B) Pb-Pb space (^206^Pb/^204^Pb vs.^208^Pb/^204^Pb and ^207^Pb/^204^Pb). Relative to local fauna, Harappa first molars have non-local distributions in (C) Pb-Sr space (^206^Pb/^204^Pb vs. ^87^Sr/^86^Sr) and (D) Pb-Pb space (^206^Pb/^204^Pb vs.^208^Pb/^204^Pb and ^207^Pb/^204^Pb).

The local isotopic ranges coincide with patterns observed in the human isotope data, further suggesting that our isotopic estimates of local dietary catchments reflect actual provisioning practices. When plotted in multi-isotopic space, some second and third molars overlap with local fauna and sediments whereas first molar cohorts plot separately from the local distributions (Fig 4A–4D). Overlap between the Harappa first molar cohort and a single faunal data point in Pb-Pb space (Fig 4D) likely represents the outermost limits of the Harappan dietary catchment and suggests our definition of local isotopic ranges may slightly overestimate the lower limit of local isotopic variation. Similarly, the Farmana third molar cohort plots between Rakhigarhi fauna and Farmana sediment leachates suggesting that our baseline data provide a conservative estimate of local ranges.

### Explaining Human Variation

**Fig 5:**
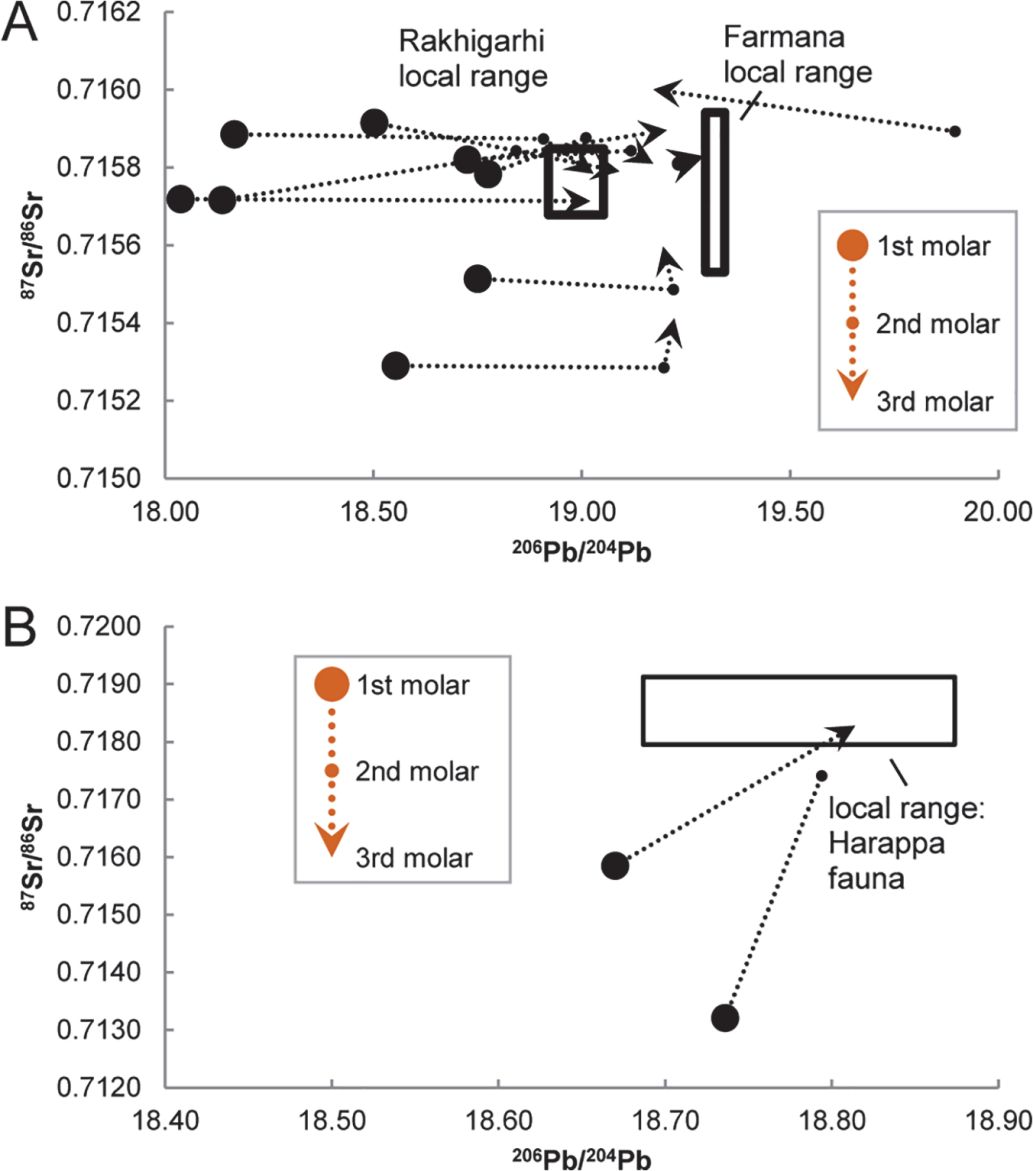
Convergence on local isotopic environments over developmental time. Heavy isotope ratio (^206^Pb/^204^Pb vs. ^87^Sr/^86^Sr) developmental series progress through early, middle, and late childhood at (A) Farmana (n = 11) and (B) Harappa (n = 3). One highly radiogenic Farmana individual is not visible at this scale, and one Harappa individual is excluded because the early-life residence change is recorded only by oxygen isotope values.

Farmana first molars fall outside the inferred local ranges of isotopic variation and are entirely separate from second and third molar distributions (Fig 4A and 4B). Likewise, most Harappa first molars are clearly distinguished from the local isotopic range (Fig 4C and 4D). Two individuals have first molars adjacent to the local range, although they are peripheral to the relatively radiogenic distribution of second and third molars that most closely match the local isotopic environment. In fact, the majority of second and third molars at Farmana and Harappa plot on a spectrum between the first molar distributions and inferred local ranges, suggesting that later developing tooth enamel in our sample recorded one or more shifts between isotopic environments during childhood. Intra-individual developmental sequences at Farmana and Harappa confirm this trend, revealing a broad convergence on local isotopic ranges during childhood. The Farmana data indicate relative stability in ^87^Sr/^86^Sr with a shift away from a non-local Pb source (or multiple Pb sources) towards local Pb ratios at approximately three to five years of age (Fig 5A). Linear trends in the developmental series consisting of three isotopically distinct teeth do not necessarily imply three different environmental sources. Rather, second molars with intermediate values most likely represent a time-averaged record of a single shift between two endpoint sources during the multi-year mineralization process. Of the three developmental series available from Harappa, two individuals display inter-tooth variation consistent with an early-life shift in the source of environmental Pb and Sr (Fig 5B). Heavy isotope ratios remain relatively static for a third individual (H94 /253 18), but a 3.8‰ decrease in stable oxygen isotope values (δ^18^O) [12] for this individual between the first and second molars suggests a marked environmental change during childhood. Several second and third molars from Harappa plot within the range of the first molars indicating they did not reach Harappa until late childhood or adulthood (Fig 4C and 4D). However, all available developmental sequences are consistent with an initial early-life shift in isotopic exposure.

Residence change between distinct geochemical environments best explains the observed isotopic differences for each individual sampled. Patterning by tooth type suggests these data reflect *in vivo* isotope ratios rather than homogenizing effects due to diagenetic alteration. Similarly, sex-based isotopic differences suggest that first molar data cannot be explained by maternal residential history. Poor preservation precludes a consideration of biological sex at Farmana, but at Harappa, male first molars have significantly lower ^87^Sr/^86^Sr compared to female first molars (*t*(14) = 1.976, *p* = 0.034), and overlapping yet distinct distributions in Pb-Pb space (S1 Fig). Furthermore, after discarding the clear radiogenic outlier in the female data set (H88/ 216 219a has higher Pb ratios than the local range), female first molars have significantly lower ^206^Pb/^204^Pb (*t*(13) = 1.986, *p* = 0.034) than male first molars. In principle, first molars identified as non-local might reflect the Pb and Sr content of breast milk consumed in the first three years of life and ultimately derived from the diet and skeleton of immigrant women [54, 55]. However, sex-structured migration more logically accounts for the observed pattern than elaborate rules of mortuary inclusion in which locally born males and females were nursed by immigrants from different regions. Furthermore, the isotopic influence of nursing on bulk enamel samples should decrease sharply in conjunction with the weaning process, the introduction of solid foods (generally having far higher Sr:Ca ratios than breast milk) [43], and the ingestion of particulate (the primary pathway for human Pb exposure) [37]. In short, age- and sex-related dietary changes are unlikely causes of the multi-isotopic shifts observed between tooth types.

One alternative to the ‘all-immigrant’ interpretation of the Harappa and Farmana mortuary samples is culturally mediated, age-specific exposure to imported sources of Pb such as that found in certain kinds of cosmetics [56]. Non-local individuals from Harappa are clearly differentiated by Pb and Sr and are therefore consistent with exposure to broadly differentiated geochemical environments. At Farmana, however, the first molar cohort is distinguished primarily by Pb isotope ratios. Low variation in ^87^Sr/^86^Sr ratios suggests regional homogenization, consistent with a shared source of aeolian sediments throughout much of the Thar Desert and adjacent semi-arid terrain [52, 57]. The lowest Pb isotope ratios, in contrast, suggest exposure to certain technological sources rather than relatively radiogenic Pb derived from the local continental crust [58]. The least radiogenic samples reflect exposure to Pb derived from minerals with low time-integrated U/Pb and Th/Pb ratios, such as the copper-rich sulfides of the historically mined Khetri region < 150 km to the south [59]. Present isotopic evidence cannot determine whether individuals lived locally and were exposed to an imported source of Pb exclusively in early childhood or whether they lived in proximity to mining and smelting activities in the hinterlands prior to their migration. However, the fact that two non-local pigs in the Rakhigarhi sample exhibit similar Pb isotope ratios to some Farmana first molars suggests that Pb exposure pathways were at the level of the local environment rather than the direct application of Pb-bearing substances such as cosmetics. Furthermore, broad isotopic and archaeological similarity with the Harappa sample suggests that a common set of practices (i.e., migration) best explains the observed isotopic pattern.

### Tracking Migration

**Fig 6:**
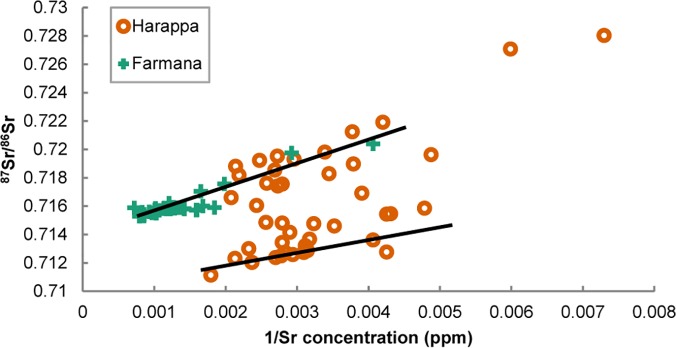
Two hypothesized Sr mixing systems based on Harappa and Farmana human data. The less radiogenic mixing system (^87^Sr/^86^Sr < ~0.716) is likely associated with Catchment A in Fig 1. The more radiogenic mixing system (^87^Sr/^86^Sr > ~0.716) is likely associated with Catchment B.

Regional geochemical assays constrain the provenience of non-local individuals. A plot of the reciprocal of Sr concentration against soluble ^87^Sr/^86^Sr reveals two mixing systems, suggesting that individuals lived in different biogeochemical catchments (Fig 6). The more radiogenic values from Harappa fall along the same mixing line as the Farmana dataset, whereas less radiogenic values (< 0.716) reflect contributions from distinct environmental sources including lower and upper end members with ^87^Sr/^86^Sr < 0.711 and > 0.716, respectively. Three end members control the Indus River Basin Sr budget [60], resulting in two convergent mixing systems that underlie the linear distributions of human data apparent in Fig 6. Carbonate weathering of the Western Fold Belt dominates the lower Indus and results in relatively low ^87^Sr/^86^Sr ratios (~0.711–0.712), but tributaries of the Punjab plains and the upper Indus channel drain distinct lithological units. Moderately radiogenic contributions (~0.712–0.716) from the Hindu Kush and Karakoram characterize upper Indus tributaries as far south as the Potwar Plateau. With the exception of the Jhelum River (~0.712–0.713), which flows adjacent to the Potwar Plateau, the Punjab tributaries drain highly radiogenic terrain (> 0.716) in the Greater and Lesser Himalayas. Therefore, Harappan individuals plotting on the less radiogenic mixing line (^87^Sr/^86^Sr < 0.716) (Fig 6) most likely originated to the northwest in the highlands and foothills stretching from the Potwar Plateau to the tributaries of the upper Indus (Catchment A). Indeed, most Harappan first molars plot along the low ^87^Sr/^86^Sr mixing line, although early-life residence was not restricted to the northwestern areas. Harappa and Farmana individuals with ^87^Sr/^86^Sr > 0.716 probably resided in the plains and foothills to the north and east where relatively radiogenic sediments of the Greater and Lesser Himalayas predominate (Catchment B).

It is difficult to define similar thresholds for Pb isotope ratios based on the geological literature because the published isotopic characterizations of ore bodies, bedrock, and sediments are not wholly representative of human exposure to biologically available Pb in a given region. Given the potential for anthropogenic Pb sources to contribute to the biologically available Pb in sediment leachates [61], isotopic ratios from base metal deposits mined in antiquity can suggest potential end members for the distributions of human isotopic ratios in Pb-Pb space. Yet the published data on ores and slags are equivocal, with potential end members located in upland terrain along the southwest coast [9, 62], near the Hindu Kush range and other parts of modern day Afghanistan [63], the Himalayan ranges to the northeast [9, 64], and different sites within the Aravallis [9, 65, 66]. Only additional samples from archaeological fauna and sediments in the surrounding regions can fully resolve the matter. Despite such limitations, the Pb isotope data from human migrants strongly implicate the Indus hinterlands known to have been the source for many of the imported minerals discovered at Harappa. One of the closest and most significant sources was the Potwar Plateau and adjacent northwestern highlands of Catchment A [9], thus suggesting a potential link between trade and migration. Indeed, Pb isotope ratios corroborate the distinction between catchments with low ^87^Sr/^86^Sr residents of Catchment A having significantly lower ^206^Pb/^204^Pb (*t*(42) = 2.713, *p* = 0.005), ^207^Pb/^204^Pb (*t*(42) = 4.544, *p* < 0.0001) and ^208^Pb/^204^Pb (*t*(42) = 2.665, *p* = 0.005) relative to the high ^87^Sr/^86^Sr residents of Catchment B. Trade and migration may have been similarly linked for residents of Farmana and the Khetri region. The Pb isotope ratios of copper slags from the sites of Singhana and Ganeshwar are the geographically closest potential end member for Farmana first molars [9], and people from the Khetri region are thought to have traded extensively with their Indus Civilization counterparts [11, 67].

### Migration as an Institution

Migrants and trade goods may have followed the same routes, but the structure of the data sets from Harappa and Farmana defies intuitive models of mortuary populations as cross sections of a breeding population or ethnic group. Over several generations, even cemeteries used by migrant communities are likely to contain a relatively large number of locally born individuals, but the isotopic data presented here suggest that an enduring minority practice of cemetery burial was limited almost entirely to first generation immigrants. The hypothetical systems of marital residence change needed to explain the isotopic data from Harappa and Farmana require multiple preconditions that appear untenable when considered collectively. Women would need to marry and reside extra-locally but return to their paternal residence for child-rearing and for their own burial. Men would also have to marry, reside, and be buried extra-locally but at different communities than their sisters. Even unwed men and boys would need to receive extra-local burial. Lastly, in order for the system to be reciprocal and sustainable, multiple communities would have to participate—communities with roughly comparable cemeteries from isotopically plausible regions that have yet to be discovered despite decades of excavation in South Asia. Indeed, well documented contemporaneous cemeteries exist at only seven Indus sites: Mehrgarh [68], Harappa [69, 70, 71, 72], Kalibangan [73], Lothal [74, 75, 76], Rakhigarhi [77, 78], Tarkhanewala-Dera [79], and Farmana [6]. None of these fall in the primary hypothesized source areas of Catchment A or the Khetri region.

Rather than trying to apply a conventional kin-based model, Harappa Phase mortuary practices may be better understood through the lens of archaeological and contemporary cemetery studies in which cemeteries are considered diverse and dynamic loci for complex cultural and political processes [23, 25, 80, 81]. Relationships between groups can be influenced by the decisions to inter different kinds of people in different ways, emphasizing various group and individual identities [82]. Importantly, group identities acquired in life can take precedence over kinship or shared local residence as with many modern military and wartime cemeteries [83]. Likewise, archaeological individuals with diverse geographic origins have been grouped in death with non-kin based on their participation in particular cultural institutions. For instance, the New York African Burial Ground [84] and Inca child sacrifice or *Capacocha* [85] are two well-known examples in which inclusion in a mortuary context was determined by the social identity of the deceased in addition to their place of residence. Therefore, the persistence over hundreds of years of all-immigrant cemeteries in the Indus context may best be interpreted as evidence for a regularized institution of immigration culminating in a distinct mode of disposal at death.

## Conclusion

Intra-individual isotopic analyses of human tooth enamel from Harappa Phase cemetery burials at Harappa and Farmana provide strong evidence that the mortuary populations were nearly entirely composed of first generation immigrants. This inference rests primarily on the use of multiple isotope systems and standard statistical methods for assessing the most plausible isotopic ranges of local dietary catchments at the study sites. Neither Sr nor Pb isotope data alone are sufficient to support the inferences presented in this paper, but their combined use reveals patterns not readily apparent when analyzing Sr alone. Beyond the increase in discriminatory power, the use of Sr and Pb isotope ratios for detecting migration benefits from the elements’ distinct pathways of incorporation into biological tissues. Whereas non-local Sr isotope ratios might be explained by imported food resources or non-local Pb isotope ratios by imported minerals, the combination of non-local Sr and Pb isotope ratios at Harappa is best explained by residence change between distinct geochemical environments. This interpretation is strengthened further by the separate origins of males and females.

In addition to patterns in the human data, statistical assessment of solutions derived from DBSCAN and K-means clustering algorithms makes it possible to identify plausible local isotopic ranges. Variation in the faunal isotope ratios is far greater than that of typical human dietary catchments in antiquity [43], suggesting that some of the sampled fauna were transported from outlying regions. By contrast, our statistically inferred local ranges exclude non-clustering data points and are consistent with typical dietary catchments as well as the specific geochemical characteristics of the study area [52, 57]. Comparison with human data also suggests that the inferred local ranges reflect actual provisioning behaviors. Local ranges overlap in Pb-Sr space with the majority of the second and third molar cohorts at Farmana and some of the third molar cohort at Harappa. All other teeth, including the first molar cohort, fall outside of the local ranges and clearly demonstrate exposure to non-local isotopic environments in early childhood. Imported goods containing Pb could potentially explain non-local isotopic ratios in the Farmana first molar cohort if exposure was specific to very young children or pregnant women, but similar Pb isotope ratios for Farmana first molars and two non-local fauna implicate a generalized environmental source. In short, residence change best explains the differences between human values and the local isotopic ranges.

Given the limitations in the sample, however, it is conceivable that non-local first molar cohorts are not representative of the entire mortuary populations. Further analyses are necessary to confirm the initial results, but the consistency with which first molars exhibit non-local isotopic ratios provides strong evidence for a class of first generation immigrants. Less certain are inferences about the timing of migration in early childhood which, at Harappa, are based only on three developmental sequences of two teeth each. Eleven developmental sequences from Farmana show a similar pattern, but it remains unclear whether or not the timing of migration can be extrapolated to the larger mortuary populations. Despite such shortcomings, the exceptionally high frequency of first generation migrations and apparent lack of locally born offspring demands a consideration of new interpretive models.

New models of the Harappa Phase cemetery mortuary program must acknowledge the enduring, institutional character of migration in the life histories of the deceased. A consideration of the isotopic data and their bioarchaeological context allows certain inferences to be made about the proposed Indus institution. Isotopic [12] and osteological [13] distinctions between males and females at Harappa and the timing of migration at Farmana suggest certain hinterland individuals from distinct genetic populations took up residence with new corporate groups at a very young age. The inclusion of modest burial wealth may indicate they were treated with respect by local groups, whereas sex-based distinctions in provenience suggest migrants were selected according to the preferences of their natal groups rather than the whims of Indus urbanites. Thus the Indus institution of immigration was integrative, accommodating various ethnic or cultural groups within a standardized set of practices reserved exclusively for a class of first generation immigrant. Furthermore, the scarcity of cemetery inhumations and their potential association with resource-rich regions suggests the institution may have been limited in scope to the economic interests of specific mercantile groups rather than all segments of society. Ethnography from the nearby Hindu Kush Range suggests one possible analogy for the Indus institution. Asymmetric systems of fosterage employed by fractious historical kingdoms to build hierarchical political alliances [86] may be broadly comparable to Indus practices, such that fostered individuals literally embodied the relationships between urban and hinterland groups. Whether or not this particular model is borne out by additional multi-disciplinary analyses, however, our isotopic inferences of migration define key parameters in any future investigation of Indus Civilization interregional interaction.

## Supporting Information

S1 Fig Differential distribution of the Harappa first molar cohort by sex (^206^Pb/^204^Pb vs.^208^Pb/^204^Pb and ^207^Pb/^204^Pb). (TIF)

S1 Table Biogeochemical data for all analyses. (DOCX)

S2 Table Minimum and maximum heavy isotope ratios for inferred local isotopic ranges of Indus Civilization sites. (DOCX)
