# Supplementary figures and images for: Evidence for Patterns of Selective Urban Migration in the Greater Indus Valley (2600-1900 BC): A Lead and Strontium Isotope Mortuary Analysis
Source: PLoS One. 2015 Apr 29;10(4):e0123103. doi: 10.1371/journal.pone.0123103 (PMC4414352; doi:10.1371/journal.pone.0123103)

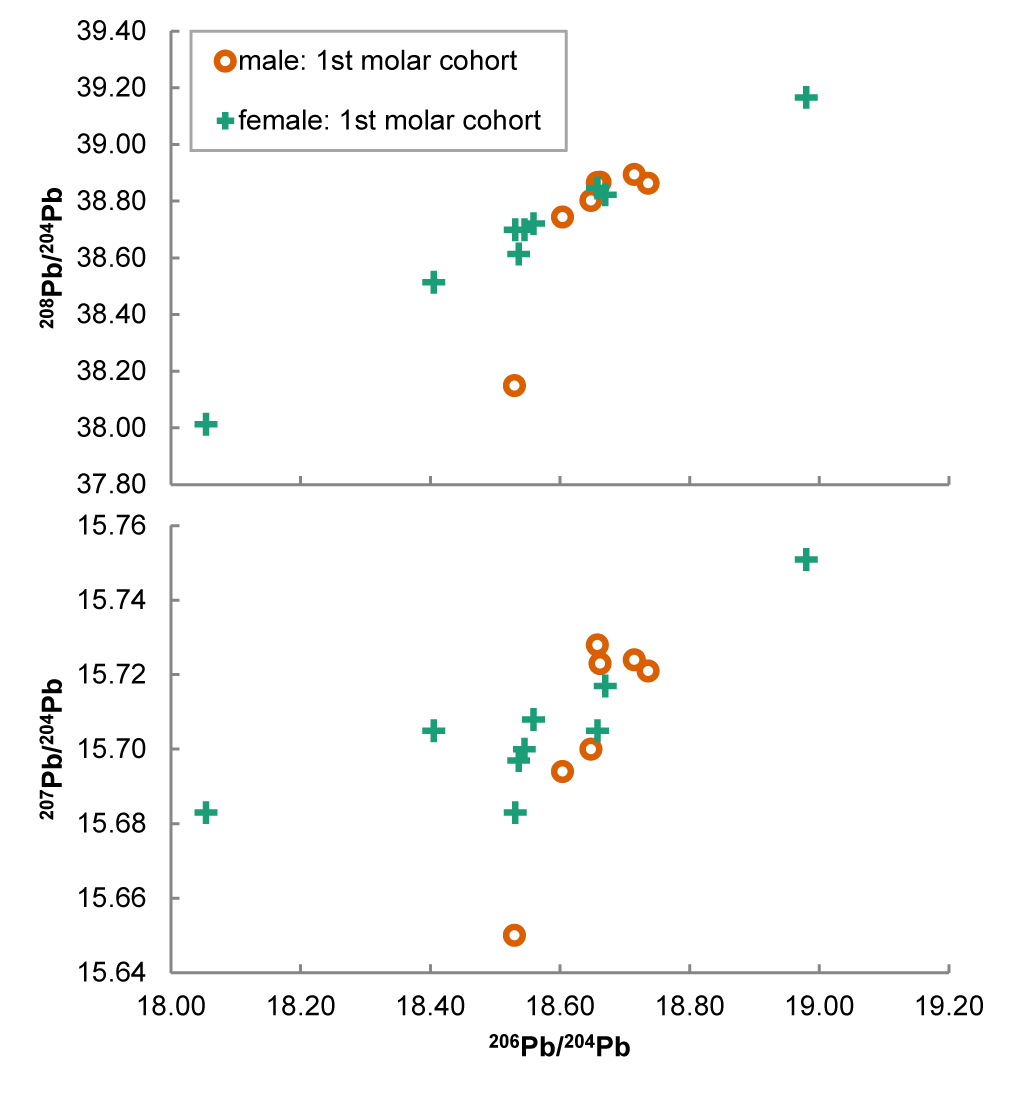

Supplement: S1 Fig — (TIF) [file pone.0123103.s001.tif]
