# Supplementary material for: Evidence for Patterns of Selective Urban Migration in the Greater Indus Valley (2600-1900 BC): A Lead and Strontium Isotope Mortuary Analysis
Source: PLoS One. 2015 Apr 29;10(4):e0123103. doi: 10.1371/journal.pone.0123103 (PMC4414352; doi:10.1371/journal.pone.0123103)
Supplement: S1 Table — (DOCX) [file pone.0123103.s002.docx]

| Burial/Specimen # | Site | Taxon/Leachate | Tooth | ^87^Sr/^86^Sr | Sr ppm | ^208^Pb/^204^Pb | ^207^Pb/^204^Pb | ^206^Pb/^204^Pb |
| --- | --- | --- | --- | --- | --- | --- | --- | --- |
| F2-1 | Farmana | *Homo* | LM_1_ | 0.71529 | 1167 | 38.623 | 15.734 | 18.553 |
| F2-2 | Farmana | *Homo* | LM_2_ | 0.71529 | 1260 | 39.351 | 15.805 | 19.197 |
| F2-3 | Farmana | *Homo* | LM_3_ | 0.71541 | 1163 | 39.401 | 15.809 | 19.222 |
| F6-1 | Farmana | *Homo* | LM_1_ | 0.71582 | 704 | 38.871 | 15.727 | 18.725 |
| F6-2 | Farmana | *Homo* | LM_2_ | 0.71584 | 778 | 39.200 | 15.775 | 19.118 |
| F6-3 | Farmana | *Homo* | LM_3_ | 0.71581 | 881 | 39.286 | 15.796 | 19.171 |
| F11-2 | Farmana | *Homo* | LM_2_ | 0.71581 | 881 | 39.351 | 15.803 | 19.233 |
| F11-3 | Farmana | *Homo* | LM_3_ | 0.71583 | 857 | 39.413 | 15.814 | 19.293 |
| F14-1 | Farmana | *Homo* | RM^1^ | 0.71551 | 1030 | 38.842 | 15.723 | 18.750 |
| F14-2 | Farmana | *Homo* | LM^2^ | 0.71549 | 971 | 39.315 | 15.786 | 19.218 |
| F14-3 | Farmana | *Homo* | RM^3^ | 0.71560 | 1072 | 39.211 | 15.783 | 19.195 |
| F18-1 | Farmana | *Homo* | LM^1^ | 0.71589 | 758 | 38.133 | 15.679 | 18.166 |
| F18-2 | Farmana | *Homo* | LM_2_ | 0.71587 | 739 | 38.929 | 15.753 | 18.908 |
| F18-3 | Farmana | *Homo* | RM_3_ | 0.71578 | 702 | 39.044 | 15.766 | 19.028 |
| F20-1 | Farmana | *Homo* | RM^1^ | 0.71572 | 626 | 37.950 | 15.673 | 18.037 |
| F20-3 | Farmana | *Homo* | RM^3^ | 0.71571 | 630 | 39.041 | 15.775 | 19.020 |
| F23-1 | Farmana | *Homo* | RM_1_ | 0.71592 | 788 | 38.546 | 15.700 | 18.502 |
| F23-2 | Farmana | *Homo* | RM_2_ | 0.71584 | 984 | 38.871 | 15.741 | 18.842 |
| F23-3 | Farmana | *Homo* | RM_3_ | 0.71579 | 816 | 39.150 | 15.775 | 19.090 |
| F26-2 | Farmana | *Homo* | RM^2^ | 0.71589 | 542 | 39.387 | 15.801 | 19.896 |
| F26-3 | Farmana | *Homo* | RM^3^ | 0.71600 | 595 | 39.184 | 15.777 | 19.172 |
| F41-1 | Farmana | *Homo* | RM^1^ | 0.71576 | 983 | 36.279 | 15.572 | 16.506 |
| F45-p3 | Farmana | *Homo* | RP^3^ | 0.71756 | 504 | 39.190 | 15.782 | 19.086 |
| F47-p4 | Farmana | *Homo* | RP^4^ | 0.71588 | 1388 | 39.148 | 15.773 | 19.075 |
| F54-1 | Farmana | *Homo* | RM_1_ | 0.71620 | 828 | 39.161 | 15.765 | 19.100 |
| F58-2 | Farmana | *Homo* | RM_2_ | 0.71570 | 933 | 38.975 | 15.744 | 18.903 |
| F62-1 | Farmana | *Homo* | LM^1^ | 0.71572 | 991 | 38.027 | 15.687 | 18.137 |
| F62-3 | Farmana | *Homo* | LM^3^ | 0.71590 | 820 | 39.280 | 15.797 | 19.204 |
| F65-1 | Farmana | *Homo* | RM_1_ | 0.71578 | 855 | 38.899 | 15.727 | 18.776 |
| F65-2 | Farmana | *Homo* | RM_2_ | 0.71588 | 814 | 39.086 | 15.757 | 19.010 |
| F66-3 | Farmana | *Homo* | RM^3^ | 0.71704 | 605 | 39.200 | 15.766 | 19.160 |
| F67-2 | Farmana | *Homo* | LM^2^ | 0.71975 | 342 | 39.228 | 15.798 | 19.299 |
| F67-3 | Farmana | *Homo* | LM_3_ | 0.72038 | 246 | 38.814 | 15.738 | 19.013 |
| H87/ 25 18a | Harappa | *Homo* | LM_1_ | 0.71485 | 390 | 38.013 | 15.683 | 18.054 |
| H87/ 40 34a | Harappa | *Homo* | LM_2_ | 0.71459 | 284 | 38.905 | 15.714 | 18.723 |
| H87/ 40 34a.2 | Harappa | *Homo* | RM_1_ | 0.71360 | 246 | 38.865 | 15.728 | 18.658 |
| H87/ 40-89 34b.1 | Harappa | *Homo* | RM_1_ | 0.71236 | 370 | 38.867 | 15.723 | 18.662 |
| H87/ 71 49b1 | Harappa | *Homo* | RM_3_ | 0.71343 | 358 | 38.672 | 15.681 | 18.566 |
| H87/ 71 49c | Harappa | *Homo* | M_1_ | 0.71585 | 209 | 38.822 | 15.717 | 18.670 |
| H87/ 71 49c | Harappa | *Homo* | M_3_ | 0.71828 | 290 | 39.030 | 15.735 | 18.814 |
| H87/ 72 49h | Harappa | *Homo* | LI_1_ | 0.71321 | 321 | 38.863 | 15.721 | 18.736 |
| H87/ 72 49h | Harappa | *Homo* | LC | 0.71741 | 366 | 39.033 | 15.732 | 18.794 |
| H87/ 72 49h | Harappa | *Homo* | M_1_ | 0.71754 | 358 | 38.894 | 15.724 | 18.715 |
| H87/ 72 49b | Harappa | *Homo* | RM^1^ | 0.71415 | 345 | 38.700 | 15.683 | 18.531 |
| H87/ 85 49g | Harappa | *Homo* | LM_2_ | 0.71922 | 404 | 38.746 | 15.712 | 18.565 |
| H87/ 85 49g | Harappa | *Homo* | LM_2_ | 0.72190 | 238 | 39.066 | 15.736 | 18.825 |
| H87/ 85 49h | Harappa | *Homo* | RM_2_ | 0.71760 | 388 | 39.081 | 15.743 | 18.868 |
| H87/ 91 80a | Harappa | *Homo* | RM^2^ | 0.71659 | 481 | 38.821 | 15.727 | 18.724 |
| H87/ 92 81a | Harappa | *Homo* | LM^3^ | 0.71817 | 457 | 38.684 | 15.713 | 18.498 |
| H87/ 108 126a | Harappa | *Homo* | RM^3^ | 0.71880 | 467 | 39.095 | 15.742 | 18.856 |
| H87/ 116 128a | Harappa | *Homo* | LM_1_ | 0.71305 | 320 | 38.731 | 15.687 | 18.621 |
| H87/ 136 147a | Harappa | *Homo* | LM_1_ | 0.71272 | 323 | 38.802 | 15.700 | 18.648 |
| H87/ 141 152a | Harappa | *Homo* | LM^1^ | 0.71962 | 205 | 38.721 | 15.708 | 18.559 |
| H87/ 145 156a | Harappa | *Homo* | LM_1_ | 0.71853 | 372 | 38.515 | 15.705 | 18.405 |
| H87/ 200 203a | Harappa | *Homo* | RM_2_ | 0.71691 | 256 | 39.377 | 15.768 | 19.099 |
| H88/ 114 127a | Harappa | *Homo* | LM_1_ | 0.71230 | 469 | 38.699 | 15.700 | 18.545 |
| H88/ 130 147a | Harappa | *Homo* | LM_2_ | 0.71202 | 422 | 39.007 | 15.725 | 18.793 |
| H88/ 162 121 | Harappa | *Homo* | RP^3^ | 0.72123 | 265 | 39.046 | 15.735 | 18.804 |
| H88/ 173 133a.10 | Harappa | *Homo* | LM_2_ | 0.71930 | 338 | 38.767 | 15.709 | 18.608 |
| H88/ 174 126b.2 | Harappa | *Homo* | RM^1^ | 0.71546 | 232 | 38.846 | 15.705 | 18.658 |
| H88/ 174 126b.2 | Harappa | *Homo* | RM^3^ | 0.71543 | 235 | 39.043 | 15.730 | 18.798 |
| H88/ 180 170a.17 | Harappa | *Homo* | LM^1^ | 0.71367 | 315 | 38.857 | 15.701 | 18.665 |
| H88/ 180 170a.17 | Harappa | *Homo* | LM^3^ | 0.71286 | 318 | 39.000 | 15.719 | 18.778 |
| H88/ 191 185c.1 | Harappa | *Homo* | RM^2^ | 0.72707 | 167 | 39.054 | 15.748 | 18.901 |
| H88/ 191 185f | Harappa | *Homo* | LM_1_ | 0.72802 | 137 | 38.827 | 15.770 | 18.845 |
| H88/ 194 196a | Harappa | *Homo* | LM_2_ | 0.71275 | 235 | 38.935 | 15.713 | 18.724 |
| H88/ 198 200a | Harappa | *Homo* | LM^2^ | 0.71980 | 295 | 38.656 | 15.690 | 18.559 |
| H88/ 201 204a | Harappa | *Homo* | RM_1_ | 0.71951 | 367 | 38.878 | 15.710 | 18.697 |
| H88/ 216 219a | Harappa | *Homo* | LM^1^ | 0.71896 | 264 | 39.167 | 15.751 | 18.980 |
| H88/ 217 220a | Harappa | *Homo* | LM_2_ | 0.71300 | 430 | 38.737 | 15.689 | 18.576 |
| H88/ 439 4b | Harappa | *Homo* | LM_2_ | 0.71258 | 340 | 38.753 | 15.710 | 18.604 |
| H94/ 243 5#2 | Harappa | *Homo* | LM^2^ | 0.71113 | 557 | 38.625 | 15.678 | 18.612 |
| H94/ 243 27 | Harappa | *Homo* | LM_2_ | 0.71604 | 411 | 38.934 | 15.735 | 18.856 |
| H94/ 245 7 | Harappa | *Homo* | LM_1_ | 0.71475 | 309 | 38.613 | 15.697 | 18.536 |
| H94/ 250 17 | Harappa | *Homo* | RM_1_ | 0.71480 | 358 | 38.744 | 15.694 | 18.604 |
| H94/ 253 18 | Harappa | *Homo* | LM_1_ | 0.71274 | 353 | 38.149 | 15.650 | 18.530 |
| H94/ 253 18 | Harappa | *Homo* | LM_2_ | 0.71248 | 360 | 38.172 | 15.650 | 18.535 |
| HA05 | Harappa | *Sus* |  | 0.71892 |  | 39.149 | 15.749 | 18.845 |
| HA06 | Harappa | *Sus* |  | 0.71869 |  | 39.189 | 15.749 | 18.858 |
| HC1 | Harappa | *Canis* |  | 0.71828 |  | 38.860 | 15.720 | 18.687 |
| HC2 | Harappa | *Canis* |  | 0.71797 |  | 39.071 | 15.733 | 18.874 |
| HC3 | Harappa | *Canis* |  | 0.71828 |  | 39.027 | 15.728 | 18.762 |
| HS1 | Harappa | *Sus* |  | 0.71913 |  | 39.042 | 15.733 | 18.779 |
| HS2 | Harappa | *Sus* |  | 0.72112 |  | 38.939 | 15.733 | 18.684 |
| HS3 | Harappa | *Sus* |  | 0.71796 |  | 38.919 | 15.730 | 18.718 |
| HS4 | Harappa | *Sus* |  | 0.72084 |  | 39.087 | 15.737 | 18.791 |
| HS5 | Harappa | *Sus* |  | 0.71569 |  | 38.818 | 15.741 | 18.674 |
| HS6 | Harappa | *Sus* |  | 0.71855 |  | 38.580 | 15.728 | 18.468 |
| HS7 | Harappa | *Sus* |  | 0.71795 |  | 38.979 | 15.736 | 18.796 |
| HS8 | Harappa | *Sus* |  | 0.71908 |  | 38.955 | 15.737 | 18.735 |
| RS1 | Rakhigarhi | *Sus* |  | 0.71574 |  | 39.054 | 15.781 | 18.971 |
| RS2 | Rakhigarhi | *Sus* |  | 0.71585 |  | 39.188 | 15.799 | 19.053 |
| RS3 | Rakhigarhi | *Sus* |  | 0.71568 |  | 38.996 | 15.781 | 18.920 |
| RS4 | Rakhigarhi | *Sus* |  | 0.71471 |  | 38.815 | 15.715 | 18.660 |
| RS5 | Rakhigarhi | *Sus* |  | 0.71903 |  | 39.070 | 15.763 | 18.970 |
| RS6 | Rakhigarhi | *Sus* |  | 0.71702 |  | 39.020 | 15.759 | 18.887 |
| RS7 | Rakhigarhi | *Sus* |  | 0.71582 |  | 39.078 | 15.765 | 19.008 |
| RS8 | Rakhigarhi | *Sus* |  | 0.71556 |  | 38.371 | 15.655 | 18.584 |
| F2Sa | Farmana | acetic |  | 0.71553 |  | 39.421 | 15.823 | 19.339 |
| F2Sh | Farmana | hydrochloric |  | 0.71565 |  | 39.452 | 15.822 | 19.341 |
| F20Sa | Farmana | acetic |  | 0.71559 |  | 39.393 | 15.817 | 19.297 |
| F20Sh | Farmana | hydrochloric |  | 0.71565 |  | 39.402 | 15.813 | 19.307 |
| F53Sa | Farmana | acetic |  | 0.71554 |  | 39.425 | 15.821 | 19.339 |
| F53Sh | Farmana | hydrochloric |  | 0.71594 |  | 39.449 | 15.82 | 19.343 |
