# Supplementary material for: Evidence for Patterns of Selective Urban Migration in the Greater Indus Valley (2600-1900 BC): A Lead and Strontium Isotope Mortuary Analysis
Source: PLoS One. 2015 Apr 29;10(4):e0123103. doi: 10.1371/journal.pone.0123103 (PMC4414352; doi:10.1371/journal.pone.0123103)
Supplement: S2 Table — (DOCX) [file pone.0123103.s003.docx]

|  | ^87^Sr/^86^Sr | | ^206^Pb/^204^Pb | |
| --- | --- | --- | --- | --- |
| Site | Min. | Max. | Min. | Max. |
| Farmana | 0.71553 | 0.71594 | 19.297 | 19.343 |
| Harappa | 0.71795 | 0.71913 | 18.687 | 18.874 |
| Rakhigarhi | 0.71568 | 0.71585 | 18.920 | 19.053 |
